# Hypotension during endovascular treatment under general anesthesia for acute ischemic stroke

**DOI:** 10.1371/journal.pone.0249093

**Published:** 2021-06-23

**Authors:** Sabine L. Collette, Maarten Uyttenboogaart, Noor Samuels, Irene C. van der Schaaf, H. Bart van der Worp, Gert Jan R. Luijckx, Allart M. Venema, Marko M. Sahinovic, Rudi A. J. O. Dierckx, Hester F. Lingsma, Teus H. Kappen, Reinoud P. H. Bokkers, MR CLEAN Registry Investigators

**Affiliations:** 1 Department of Radiology, Medical Imaging Center, University Medical Center Groningen, University of Groningen, Groningen, The Netherlands; 2 Department of Neurology, University Medical Center Groningen, University of Groningen, Groningen, The Netherlands; 3 Department of Neurology, Erasmus Medical Center, Rotterdam, The Netherlands; 4 Department of Radiology and Nuclear Medicine, Erasmus Medical Center, Rotterdam, The Netherlands; 5 Department of Public Health, Erasmus Medical Center, Rotterdam, The Netherlands; 6 Department of Radiology, University Medical Center Utrecht, Utrecht, The Netherlands; 7 Department of Neurology and Neurosurgery, University Medical Center Utrecht, Utrecht, The Netherlands; 8 Department of Anesthesiology, University Medical Center Groningen, University of Groningen, Groningen, The Netherlands; 9 Department of Nuclear Medicine and Molecular Imaging, Medical Imaging Center, University Medical Center Groningen, University of Groningen, Groningen, The Netherlands; 10 Department of Anesthesiology, University Medical Center Utrecht, Utrecht, The Netherlands; Seoul National University Hospital, REPUBLIC OF KOREA

## Abstract

**Objective:**

The effect of anesthetic management (general anesthesia [GA], conscious sedation, or local anesthesia) on functional outcome and the role of blood pressure management during endovascular treatment (EVT) for acute ischemic stroke is under debate. We aimed to determine whether hypotension during EVT under GA is associated with functional outcome at 90 days.

**Methods:**

We retrospectively collected data from patients with a proximal intracranial occlusion of the anterior circulation treated with EVT under GA. The primary outcome was the distribution on the modified Rankin Scale at 90 days. Hypotension was defined using two thresholds: a mean arterial pressure (MAP) of 70 mm Hg and a MAP 30% below baseline MAP. To quantify the extent and duration of hypotension, the area under the threshold (AUT) was calculated using both thresholds.

**Results:**

Of the 366 patients included, procedural hypotension was observed in approximately half of them. The occurrence of hypotension was associated with poor functional outcome (MAP <70 mm Hg: adjusted common odds ratio [acOR], 0.57; 95% confidence interval [CI], 0.35–0.94; MAP decrease ≥30%: acOR, 0.76; 95% CI, 0.48–1.21). In addition, an association was found between the number of hypotensive periods and poor functional outcome (MAP <70 mm Hg: acOR, 0.85 per period increase; 95% CI, 0.73–0.99; MAP decrease ≥30%: acOR, 0.90 per period; 95% CI, 0.78–1.04). No association existed between AUT and functional outcome (MAP <70 mm Hg: acOR, 1.000 per 10 mm Hg*min increase; 95% CI, 0.998–1.001; MAP decrease ≥30%: acOR, 1.000 per 10 mm Hg*min; 95% CI, 0.999–1.000).

**Conclusions:**

Occurrence of procedural hypotension and an increase in number of procedural hypotensive periods were associated with poor functional outcome, whereas the extent and duration of hypotension were not. Randomized clinical trials are needed to confirm our hypothesis that hypotension during EVT under GA has detrimental effects.

## Introduction

Endovascular treatment (EVT) is a highly effective treatment for acute ischemic stroke due to large vessel occlusion [1–6]. Nevertheless, approximately 55% of the patients are still dependent in activities of daily living or have died at 3 months after EVT [6].

EVT can be performed under general anesthesia (GA), conscious sedation (CS), or with local anesthesia (LA) at the site of puncture. The most optimal anesthetic approach during EVT is still a matter of debate since literature shows conflicting results regarding functional outcome [7–15].

One of the differences between GA, CS, and LA concerns procedural hemodynamics. In GA, and to a lesser extent CS, hypotensive periods are relatively common due to administration of anesthetic agents. In LA, these potential detrimental episodes occur less often as anesthetics are avoided. Relevant hemodynamic changes will however be noticed and might therefore be controlled more quickly during GA compared with CS and LA because of the close monitoring.

In three single-center, randomized controlled trials, the modified Rankin Scale (mRS) score at 90 days was compared between patients who were treated under GA or with CS [7–9]. Two trials showed a more favorable outcome in the GA-arm [7, 8], whereas the third found no differences between both anesthetic approaches [9]. A recent meta-analysis of these trials found a better functional outcome when EVT was performed under GA [10]. Observational studies, summarized in two meta-analyses, on the other hand, revealed better functional outcomes in the non-GA arm (CS, LA, or a combination of both) compared with the GA arm [11–14].

The discrepancies in functional outcome of patients who were treated under GA might be explained by the extent and duration of procedural hypotension depending on hemodynamic management. As in the randomized controlled trials strict protocols for anesthetic blood pressure management were followed, hypotensive periods, and poor functional outcome as a possible consequence, might have been prevented [7–9]. Most of the non-randomized controlled studies did not report procedural blood pressures or the use of protocols for periprocedural blood pressure management [11–14]. It is therefore unknown to what extent hypotension occurs in clinical practice and whether hypotension is associated with functional outcome. In addition, the non-GA arm of one meta-analysis and both observational studies might have consisted of more patients receiving LA than CS, preventing hemodynamic instability caused by anesthetic agents in patients treated under CS [11, 12, 14].

In this retrospective, observational study, we aimed to investigate whether hypotension during EVT under GA is associated with functional outcome and risk of complications after EVT in clinical practice.

## Methods

This was a retrospective, observational study of patients treated at two centers; the University Medical Center Groningen (UMCG), between December 2008 and December 2017, and the University Medical Center Utrecht (UMCU), between April 2014 and April 2017. Start of the inclusion periods differed because EVT data were not being stored systemically in the UMCU before April 2014. Both centers serve as a regional comprehensive stroke center and, during the enrollment period, patients were treated under GA as standardized primary anesthetic approach.

The study was reviewed by the Institutional Ethical Review Board of the UMCG and approval was waived (reference number: METc 2017/622) as retrospective, observational studies do not fall under the scope Medical Research Involving Human Subject Act (WMO, the Netherlands). In accordance with national privacy laws during the conduct of the study, informed consent was not required. We consulted the UMCG objections register to inquire whether patients either verbally or in writing refused the usage of their data for research purposes. All study protocols and procedures were conducted in accordance with the Declaration of Helsinki.

Data will not be made available to other researchers as no patient approval has been obtained for sharing coded data. Syntax files will be made available from the Loket Contract Research, section of the UMCG’s Department of Legal Affairs, on reasonable request (email address: loket_contract_research@umcg.nl).

### Participants

We included patients who underwent EVT under GA due to an occlusion in the intracranial carotid artery (ICA), middle cerebral artery (M1, first segment, or M2, second segment), or anterior cerebral artery (A1, first segment, or A2, second segment), confirmed by computed tomography (CT), and who had a National Institutes of Health Stroke Scale (NIHSS) score of ≥2. EVT had to be initiated within 6 hours after onset of the first symptoms.

Exclusion criteria for this study were intubation before arrival at the angiography suite, initiation of GA after start of the intervention, a procedural intracerebral hemorrhage, spontaneous recanalization on the initial digital subtraction angiography run, and technically not feasible EVT, for example due to tortuous arteries or elongation of the aortic arch.

### Endovascular procedure

Both hospitals performed EVT under GA as part of standard clinical care. Procedural blood pressure was managed according to the Society of Neuroanesthetic and Critical Care guideline. Therefore, it was attempted to maintain the systolic blood pressure (SBP) between 140 and 180 mm Hg, and the diastolic blood pressure (DBP) <105 mm Hg [16]. EVT was done with primary aspiration, stent retriever, or a combination of both techniques. The choice for the EVT device was left to the discretion of the interventionist.

Application of local urokinase or alteplase was allowed during EVT. In case of high-grade proximal carotid artery stenosis or occlusion, percutaneous transluminal angioplasty, with or without carotid artery stenting, was performed (according to the discretion of the interventionist).

Extubation was performed as early as possible after completion of the intervention. Independent of their (mechanical) ventilation status, patients were all admitted to the intensive care or stroke unit for post-intervention monitoring.

### Data collection

Clinical and imaging characteristics were retrieved from non-anonymized EVT registries at both centers. Missing clinical items and hemodynamic parameters were obtained from the patient records. Missing imaging characteristics of patients treated after March 2014 were acquired from the MR CLEAN Registry that included coded data [17]. Data were stored and analyzed coded.

#### Hemodynamic parameters

Periprocedural hemodynamic values were acquired from 5 minutes before start of induction of GA until extubation in the angiography suite (if not applicable, the end of the intervention, defined as closure of the groin puncture site). The SBP, DBP (both either non-invasive or using an intra-arterial catheter), and heart rate were recorded every 1 or every 5 minutes (UMCU and UMCG, respectively). These intervals differed because of the method of registration: handwritten anesthesia records were used in the UMCG and digital records were used in the UMCU (AnStat, CarePoint, the Netherlands).

The mean arterial pressure (MAP) 5 minutes before induction was set as a baseline value. Hypotension was defined using 2 thresholds because of a lack of a generally accepted definition of hypotension in patients with acute ischemic stroke [18]. They were set as (1) an absolute MAP threshold of 70 mm Hg and (2) a relative threshold of a MAP 30% below baseline MAP [19–21]. Subsequently, hypotension was quantified as (A) an area under the threshold (AUT; expressed in 10 mm Hg below the threshold*min) to summarize the extent and duration of a blood pressure decrease (S1 Fig), (B) occurrence of hypotension, (C) number of hypotensive periods, and (D) total hypotension duration (in minutes). In addition to hypotension, a decrease in blood pressure was analyzed. This was quantified as the difference between the baseline MAP and the single lowest procedural MAP (ΔMAP) (S1 Fig).

### Outcomes

The primary outcome was the distribution on the mRS at 90 days after the procedure, a categorical scale (0, no symptoms; 6, death) that displays the degree of disability after a stroke [22].

Secondary (safety) outcomes were successful reperfusion, early neurologic recovery, symptomatic intracranial hemorrhage (sICH) within 48 hours after the procedure and in-hospital mortality. Reperfusion was assessed with the modified Thrombolysis In Cerebral Infarction score (range 0 to 3; the higher the number, the greater degree of reperfusion) after intervention, with a score of ≥2B interpreted as a successful technical result and adequate reperfusion [23]. Early neurologic recovery was assessed with a postprocedural NIHSS score within 24 hours. A score of 0 or 1, or a decrease of 8 points relative to baseline, was defined as early neurologic recovery [3]. sICH was defined as parenchymal hemorrhage with early neurologic deterioration (an increase of ≥4 points in score on the NIHSS) [1].

### Statistical analysis

Continuous baseline characteristics were visually assessed for normality using histograms and quantile-quantile plots. Continuous normally distributed data are reported as the mean (SD) and were compared with the independent-sample *t* test. Continuous non-normally distributed data are reported as the median and interquartile range (IQR) and were compared with the Mann-Whitney *U* test. Categorical data are reported as number (%) and were compared with the χ^2^ test.

For both hypotension thresholds (1–2), we studied the association between the four quantifications of hypotension (A-D) and the distribution on the mRS at 90 days. Also, the association between ΔMAP and the distribution on the mRS at 90 days was analyzed. The associations were expressed as adjusted common odds ratios (acORs) derived from multivariable ordinal logistic regression analyses. To adjust for baseline prognostic factors, the model included age, sex, medical history of atrial fibrillation, diabetes mellitus, hypertension, myocardial infarction, and previous stroke, prestroke mRS score, collateral score, NIHSS score at baseline, and time from symptom onset to groin puncture.

In all analyses concerning functional outcome at 90 days, mRS scores were reversed, as this more clearly displays a shift toward a better mRS score. This means an (ac)OR >1 reflects a shift toward better functional outcome. Conversely, an (ac)OR <1 reflects a shift toward a worse functional outcome.

Associations between the quantifications of hypotension and secondary outcomes were analyzed using binary logistic regression analyses. We adjusted for the same covariates as in the primary analyses.

Missing data were imputed using multiple imputations by chained equations based on relevant covariates and outcome. The Last-Observation-Carried-Forward method was used to interpolate missing MAPs. Restricted cubic splines were used to test for nonlinearity.

All *P* values are 2 sided, and *P* < .05 was considered significant. Analyses were performed using R 3.6.1 software (The R foundation for Statistical Computing, Vienna, Austria) and SPSS Statistics 23.0 software (IBM Corp, Armonk, NY).

## Results

Between December 2008 and December 2017 (UMCG) and April 2014 and April 2017 (UMCU), 537 patients with an acute ischemic stroke and large vessel occlusion were treated with EVT. Of these 537 patients, 5 patients objected to the use of their data, 6 had an isolated M3 occlusion, 67 had an occlusion in the posterior circulation, 10 were not treated under GA (due to unavailability of an anesthesia care team while the patient was cooperative, a patient’s pre-stroke condition combined with advanced age, or unknown reasons), and 83 were excluded for various reasons. This left 366 patients to be included in this study (S2 Fig).

### Characteristics

#### Baseline characteristics

Median age was 70 (IQR, 59–78) years, and 202 patients (56%) were male (Table 1; S1 and S2 Tables).

**Table 1:** Baseline characteristics.

Characteristics	Value
Age, *median (IQR)*, years	70 (59–78)
Male, *n (%)*	202/366 (56)
Medical history, *n (%)*	
Atrial fibrillation	81/359 (22.6)
Diabetes mellitus	52/365 (14.2)
Hypercholesterolemia	180/364 (49.5)
Hypertension	221/364 (60.7)
Myocardial infarction	43/357 (12.0)
Previous stroke	45/364 (12.4)
Antithrombotic medication, *n (%)*	160/363 (44.1)
Prestroke mRS score >2, *n (%)*	35/265 (13.2)
Location occlusion, *n (%)*^a^	
Left hemisphere	204/363 (56.2)
ICA-C	60/362 (16.6)
ICA-T	95/362 (26.2)
M1	256/362 (70.7)
M2	134/362 (37.0)
A1	14/362 (3.9)
A2	9/362 (2.5)
Collateral score, *n (%)*	
Absent collaterals	31/348 (8.9)
>0% and ≤50% filling of the occluded area	145/348 (41.6)
>50% and <100% filling of the occluded area	119/348 (34.2)
100% filling of the occluded area	53/348 (15.2)
NIHSS score, *median (IQR)*	16 (12–20)
Intravenous thrombolysis, *n (%)*	254/364 (69.8)
Preintervention MAP, *mean (SD)*, mm Hg	108 (19)
Time from stroke onset to groin puncture, *median (IQR)*, minutes	216 (180–264)

A1, anterior cerebral artery, first segment; A2, anterior cerebral artery, second segment; ICA-C, cervical internal carotid artery; ICA-T, internal carotid artery terminus; M1, middle cerebral artery, first segment; M2, middle cerebral artery, second segment; MAP, mean arterial pressure; mRS, modified Rankin Scale; NIHSS, National Institutes of Health Stroke Scale.

IQR, interquartile range; n, number; SD, standard deviation.

^a^Sum may not equal 100% due to combined occlusions.

#### Procedural hemodynamic variables

For both thresholds, hypotension occurred in approximately half of the patients (MAP <70 mm Hg: 44%; MAP decrease ≥30%: 53%). Distributions of AUT were right-skewed. Median AUT was 0 (IQR, 0–35) mm Hg*min for the absolute threshold and 5 (IQR, 0–96) mm Hg*min for the relative threshold. The mean ΔMAP was 35 mm Hg (standard deviation, 18) (Table 2).

**Table 2:** Procedural variables.

Variable	Value
Thrombectomy approach, *n (%)*	
Stent retriever	22/359 (6.1)
Aspiration	113/359 (31.5)
Stent retriever and aspiration	224/359 (62.4)
Urokinase or alteplase	33/366 (9.0)
Carotid PTA	61/358 (17.0)
Carotid stenting	13/366 (3.6)
Total number of passes, *n (%)*	
1	96/236 (40.7)
2	61/236 (25.8)
3	31/236 (13.1)
≥4	48/236 (20.3)
Procedural MAP, *mean (SD)*, mm Hg	91 (14)
Occurrence of hypotension, *n (%)*	
Absolute threshold^a^	153/351 (43.6)
Relative threshold^b^	172/328 (52.4)
Number of hypotensive periods, *n (%)*	
Absolute threshold^a^	
1	88/351 (25.1)
2	30/351 (8.5)
≥3	35/351 (10.0)
Relative threshold^b^	
1	80/328 (24.4)
2	40/328 (12.2)
≥3	52/328 (15.9)
Hypotension duration, *median (IQR)*, minutes	
Absolute threshold^a^	0 (0–6)
Relative threshold^b^	3 (0–15)
Area under the threshold, *median (IQR)*, mm Hg*min	
Absolute threshold^a^	0 (0–35)
Relative threshold^b^	5 (0–96)
ΔMAP, *mean (SD)*, mm Hg^c^	35 (18)
Time from stroke onset to reperfusion, *median (IQR)*, minutes	270 (225–316)

MAP, mean arterial pressure; PTA, percutaneous transluminal angioplasty.

IQR, interquartile range; n, number; SD, standard deviation.

^a^Threshold was set to a mean arterial pressure value of 70 mm Hg.

^b^Threshold was set to a mean arterial pressure value of 30% below baseline mean arterial pressure.

^c^ΔMAP was defined as the difference between baseline MAP and single lowest procedural MAP.

### Outcome data

#### Primary outcome

Distributions of postprocedural mRS scores at 90 days were equal for patients with and without procedural hypotension (S3 Fig).

#### Absolute threshold

Both occurrence of hypotension and number of hypotensive periods were associated with poor functional outcome (acOR, 0.57; 95% confidence interval [CI], 0.35–0.94, and acOR, 0.85 per period increase; 95% CI, 0.73–0.99, respectively). AUT was not associated with functional outcome (acOR, 1.000 per 10 mm Hg*min increase; 95% CI, 0.998–1.001), neither in a subgroup analysis of patients with procedural hypotension (defined as AUT >0; acOR, 1.001 per 10 mm Hg*min; 95% CI, 0.998–1.003). Correspondingly, hypotension duration was not related to functional outcome (Table 3; S3 Table).

**Table 3:** Association of procedural hemodynamics with functional outcome.

Variable	acOR^a^,^b^	95% CI
Area under the threshold^c^		
Absolute threshold^d^	1.000	0.998–1.001
Relative threshold^e^	1.000	0.999–1.000
Occurrence of hypotension		
Absolute threshold^d^	0.57	0.35–0.94
Relative threshold^e^	0.76	0.48–1.21
Number of hypotensive periods^f^		
Absolute threshold^d^	0.85	0.73–0.99
Relative threshold^e^	0.90	0.78–1.04
Total hypotension duration^g^		
Absolute threshold^d^	0.99	0.97–1.00
Relative threshold^e^	0.99	0.98–1.00
ΔMAP^h^	0.991	0.980–1.003

MAP, mean arterial pressure.

acOR, adjusted common odds ratio; CI, confidence interval.

^a^Results were adjusted for age, sex, medical history of atrial fibrillation, diabetes mellitus, hypertension, myocardial infarction, and previous stroke, prestroke modified Rankin Scale score, collateral score, National Institutes of Health Stroke Scale score at baseline, and time from symptom onset to groin puncture.

^b^Median modified Rankin Scale score was 3 (2–6).

^c^Adjusted common odds ratio per 10 mm Hg*min increase.

^d^Threshold was set to a mean arterial pressure value of 70 mm Hg.

^e^Threshold was set to a mean arterial pressure value of 30% below baseline mean arterial pressure.

^f^Adjusted common odds ratio per period increase.

^g^Adjusted common odds ratio per minute increase.

^h^ΔMAP was defined as the difference between baseline MAP and single lowest procedural MAP.

#### Relative threshold

AUT was not associated with functional outcome (acOR, 1.000 per 10 mm Hg*min; 95% CI, 0.999–1.000), neither in a subgroup analysis of patients with procedural hypotension (acOR, 0.999 per 10 mm Hg*min; 95% CI 0.998–1.001). In addition, no association was found between the three other measures of hypotension and functional outcome (Table 3; S3 Table).

#### Lowest mean arterial pressure

In the univariable regression analysis, an association between ΔMAP and poor functional outcome was found (cOR, 0.987; 95% CI, 0.977–0.998). The association did however no longer exist after adjustment for confounders (acOR, 0.991; 95% CI, 0.980–1.003) (Table 3; S3 Table).

#### Secondary outcomes

306 of 365 patients (84%) had successful reperfusion, 85 of 347 (24%) showed early neurologic recovery, sICH was seen in 24 of 366 patients (6.6%), and 36 of 366 patients (9.8%) died during hospital admission (S4 and S5 Tables). AUT was not associated with any of the secondary outcomes (Table 4; S6 Table).

**Table 4:** Association between area under the threshold and secondary safety outcomes.

	Successful reperfusion^a^	Early neurologic recovery^b^	Symptomatic intracranial hemorrhage^c^	In-hospital mortality
*Absolute threshold*^d^				
**acOR**^e^,^f^	0.999	1.001	1.001	1.001
**95% CI**	0.997–1.002	0.998–1.002	0.998–1.004	0.999–1.004
*Relative threshold*^g^				
**acOR**^e^,^f^	1.000	1.000	1.001	1.001
**95% CI**	0.998–1.000	0.999–1.001	1.001–1.004	1.000–1.002

acOR, adjusted common odds ratio; CI, confidence interval.

^a^Successful reperfusion was defined as modified Thrombolysis In Cerebral Infarction score of ≥2B.

^b^Early neurologic recovery was defined as National Institutes of Health Stroke Scale score of 0 or 1 within 24 hours postprocedural, or a decrease of 8 points relative to baseline.

^c^Symptomatic intracranial hemorrhage was defined as parenchymal hemorrhage with early neurologic deterioration (an increase of ≥4 points in score on the National Institutes of Health Stroke Scale).

^d^Threshold was set to a mean arterial pressure value of 70 mm Hg.

^e^Results were adjusted for age, sex, medical history of atrial fibrillation, diabetes mellitus, hypertension, myocardial infarction, and previous stroke, prestroke modified Rankin Scale score, collateral score, National Institutes of Health Stroke Scale score at baseline, and time from symptom onset to groin puncture.

^f^Adjusted common odds ratio per 10 mm Hg*min increase.

^g^Threshold was set to a mean arterial pressure value of 30% below baseline mean arterial pressure.

## Discussion

Among patients treated with EVT under GA for acute ischemic stroke, we found that both occurrence of procedural hypotension and an increase in number of procedural hypotensive periods defined by the absolute threshold were associated with poor functional outcome, whereas the extent of hypotension and duration were not.

Current guidelines on hemodynamic management during EVT are based on IVT studies and expert opinions due to lack of data. It is recommended to maintain the blood pressure ≤185/110 mm Hg during EVT [24]. Despite the suggested target for a lower limit of SBP 140 mm Hg (which is also maintained by the randomized controlled trials that compared GA and CS), the definite optimal lower threshold is unknown due to lack of a clear consensus on quantification of procedural hypotension [7–9, 16, 18, 24]. This uncertainty makes it difficult to investigate the clinical effect of hypotension. In addition, EVT is a relatively new treatment option and data on periprocedural blood pressure management is scarce. Various definitions for hypotension are used in literature, each emphasizing different characteristics [18]. It is therefore difficult to compare our results with the studies mentioned below.

In a retrospective cohort study including 3 randomized clinical trials (SIESTA [Sedation vs Intubation for Endovascular Stroke Treatment], ANSTROKE [Anesthesia During Stroke], and GOLIATH [General or Local Anesthesia in Intra-Arterial Thrombectomy]), the effect of a procedural MAP below and above certain thresholds on functional outcome was analyzed. Approximately half of the 365 included patients were treated under GA, and the other half under CS. A MAP below 70 mm Hg for more than 10 minutes was found to be associated with poor functional outcome [25]. In a post-hoc analysis of the SIESTA study alone, no association between procedural hemodynamics and functional outcome was found, neither when GA and CS were analyzed separately. This might be the result of a relatively small study population including 73 patients being treated under GA and 77 patients being treated under CS [26]. Petersen et al. [27] performed a retrospective, observational study to investigate the clinical effect of a decrease in blood pressure in 390 patients who underwent EVT. GA was only used in patients that could not cooperate with the procedure despite conscious sedation, had respiratory failure, or were unable to protect their airway. Decrease in blood pressure was defined as the single greatest procedural MAP decrease from baseline, and the area between baseline MAP and continuous procedural MAP measurements. Increase of both measurements were found to be associated with poor functional outcome. Treurniet et al. [28] performed a retrospective study from the MR CLEAN trial including 85 patients who underwent EVT under GA. Decrease in blood pressure was defined as the single greatest procedural MAP decrease from baseline, and the difference between MAP as baseline and mean procedural MAP. An increased difference between baseline and mean procedural MAP was associated with worse functional outcome. Corresponding to our results, the single greatest procedural MAP decrease from baseline was not found to be associated with functional outcome.

In our study, only two of the hemodynamic measures were found to be associated with poor functional outcome. As a consequence, no clear statement can be made about a possible negative effect of low blood pressure. Associations between the other hemodynamic measures and functional outcome might however have been missed due to two reasons. First, the relatively low incidence of procedural hypotension could have led to an underpowered analysis. It seems that anesthesiologists were aware of the possible harmful effect of low blood pressure on a vulnerable brain, resulting in prevention of hypotension. Second, study results might have been confounded by indication. Patients with a worse pre- and consequently post-interventional condition could have been susceptible for hemodynamic instability during GA, for example due to a stronger effect of anesthetic agents. It seems that anesthesiologists anticipated this situation, because of the relatively moderate extent and short duration of hypotension. This might explain the discrepant significance between occurrence of hypotension and increased number of hypotensive periods compared with extent and duration of hypotension.

Hypotension was not found to be associated with an increased risk of sICH. We could not compare our results with other studies as these data were not reported. An underlying mechanism could have been a larger infarct core volume and subsequent increased hemorrhagic risk [29]. On the other hand, as hypertension is associated with sICH, an increased sICH risk could have been found in the non-hypotensive group due to increased blood pressure induced by vasopressive agents [30, 31]. Further analysis of this association was however beyond the scope of this study.

This study has several limitations. First, the baseline blood pressure was based on a single measurement, which increased the risk of a measurement error. In addition, physical stress elevates baseline blood pressure and, therefore, does not correspond with a patient’s average, home-situated blood pressure.

Second, in one center, periprocedural blood pressures were recorded in hand written anesthesia records. This may potentially have resulted in less accurate values. Moreover, hemodynamics were measured in relatively large intervals of 5 minutes. To optimize fitting of the curve, a smoothing technique was used, which may have affected the AUT.

Third, the influence of infarct location and infarct size could not be determined as these data were not available. This might have led to confounding with the likely direction in favor of the patients without procedural hypotension.

Finally, due to the retrospective study design, no study specific anesthetic protocol for blood pressure regulation and pharmacologic guidelines was maintained. Hemodynamics were managed according to recommendations of the Society for Neuroscience in Anesthesiology and Critical Care guideline [16]. As a result, incidence of hypotension was low compared with studies investigating a more general anesthesia population [20]. Consequently, association between the extent and duration of hypotension related to functional outcome could have been missed. In addition, the effect of lower thresholds on outcome could not be investigated to determine whether the currently advised targets could be lowered.

## Conclusions

Occurrence of procedural hypotension and an increase in number of procedural hypotensive periods were associated with poor functional outcome, whereas the extent and duration of hypotension were not. Randomized clinical trials are needed to confirm our hypothesis that hypotension during EVT under GA has detrimental effects.

## Supporting information

S1 Table Baseline characteristics of patients with and without hypotension. A1, anterior cerebral artery, first segment; A2, anterior cerebral artery, second segment; ICA-C, cervical internal carotid artery; ICA-T, internal carotid artery terminus; M1, middle cerebral artery, first segment; M2, middle cerebral artery, second segment; MAP, mean arterial pressure; mRS, modified Rankin Scale; NIHSS, National Institutes of Health Stroke Scale. IQR, interquartile range; n, number; SD, standard deviation. ^a^Threshold was set to a mean arterial pressure value of 70 mm Hg. ^b^Sum may not equal 100% due to combined occlusions.
(PDF)

S2 Table Baseline characteristics of patients with and without hypotension. A1, anterior cerebral artery, first segment; A2, anterior cerebral artery, second segment; ICA-C, cervical internal carotid artery; ICA-T, internal carotid artery terminus; M1, middle cerebral artery, first segment; M2, middle cerebral artery, second segment; MAP, mean arterial pressure; mRS, modified Rankin Scale; NIHSS, National Institutes of Health Stroke Scale. IQR, interquartile range; n, number; SD, standard deviation. ^a^Threshold was set to a mean arterial pressure 30% below baseline mean arterial pressure. ^b^Sum may not equal 100% due to combined occlusions.
(PDF)

S3 Table Unadjusted odds ratios for the association between procedural haemodynamics and functional outcome. CI, confidence interval; OR, odds ratio. ^a^Median modified Rankin Scale score was 3 (2–6). ^b^Odds ratio per 10 mm Hg*min increase. ^c^Threshold was set to a mean arterial pressure value of 70 mm Hg. ^d^Threshold was set to a mean arterial pressure 30% below baseline mean arterial pressure. ^e^Odds ratio per period increase. ^f^Odds ratio per minute increase. ^h^ΔMAP was defined as the difference between baseline MAP and single lowest procedural MAP.
(PDF)

S4 Table Postprocedural outcome variables of patients with and without hypotension. ^a^Threshold was set to a mean arterial pressure value of 70 mm Hg. ^b^Successful reperfusion was defined as modified Thrombolysis In Cerebral Infarction score of ≥2B. ^c^Early neurologic recovery was defined as National Institutes of Health Stroke Scale score of 0 or 1 within 24 hours postprocedural, or a decrease of 8 points relative to baseline. ^d^Symptomatic intracranial hemorrhage was defined as parenchymal hemorrhage with early neurologic deterioration (an increase of ≥4 points in score on the National Institutes of Health Stroke Scale).
(PDF)

S5 Table Postprocedural outcome variables of patients with and without hypotension. ^a^Threshold was set to a mean arterial pressure 30% below baseline mean arterial pressure. ^b^Successful reperfusion was defined as modified Thrombolysis In Cerebral Infarction score of ≥2B. ^c^Early neurologic recovery was defined as National Institutes of Health Stroke Scale score of 0 or 1 within 24 hours postprocedural, or a decrease of 8 points relative to baseline. ^d^Symptomatic intracranial hemorrhage was defined as parenchymal hemorrhage with early neurologic deterioration (an increase of ≥4 points in score on the National Institutes of Health Stroke Scale).
(PDF)

S6 Table Unadjusted odds ratios for the association between area under the threshold and secondary safety endpoints. CI, confidence interval; OR, odds ratio. ^a^Successful reperfusion was defined as modified Thrombolysis In Cerebral Infarction score of ≥2B. ^b^Early neurologic recovery was defined as National Institutes of Health Stroke Scale score of 0 or 1 within 24 hours postprocedural, or a decrease of 8 points relative to baseline. ^c^Symptomatic intracranial hemorrhage was defined as parenchymal hemorrhage with early neurologic deterioration (an increase of ≥4 points in score on the National Institutes of Health Stroke Scale). ^d^Threshold was set to a mean arterial pressure value of 70 mm Hg. ^e^Odds ratio per 10 mm Hg*min increase. ^f^Threshold was set to a mean arterial pressure 30% below baseline mean arterial pressure.
(PDF)

S1 Fig Graphical representation of the area under the threshold and ΔMAP. The dark blue line represents continuously measured mean arterial pressure (MAP) during endovascular treatment. Area under the threshold (A) was calculated as the total area between (1) an absolute threshold of MAP 70 and procedural MAP, and (2) baseline MAP and procedural MAP of 30% below baseline MAP. ΔMAP (B) was calculated as the MAP at baseline minus the lowest single MAP during endovascular treatment.
(TIF)

S2 Fig Flowchart of included patients. CS, conscious sedation; EVT, endovascular treatment; GA, general anesthesia; LA, local anesthesia; M3, middle cerebral artery, third segment. ^a^Defined as an impossibility of reaching the intracerebral occlusion (mostly due to tortuous arteries or elongation of the aortic arch).
(TIF)

S3 Fig Functional outcome at 90 days postprocedural of patients with and without hypotension. mRS, modified Rankin Scale. Number of patients are displayed as percentages.
(TIF)
