# Supplementary material for: Hypotension during endovascular treatment under general anesthesia for acute ischemic stroke
Source: PLoS One. 2021 Jun 23;16(6):e0249093. doi: 10.1371/journal.pone.0249093 (PMC8221480; doi:10.1371/journal.pone.0249093)
Supplement: S1 Table — A1, anterior cerebral artery, first segment; A2, anterior cerebral artery, second segment; ICA-C, cervical internal carotid artery; ICA-T, internal carotid artery terminus; M1, middle cerebral artery, first segment; M2, middle cerebral artery, second segment; MAP, mean arterial pressure; mRS, modified Rankin Scale; NIHSS, National Institutes of Health Stroke Scale. IQR, interquartile range; n, number; SD, standard deviation. aThreshold was set to a mean arterial pressure value of 70 mm Hg. bSum may not equal 100% due to combined occlusions. (PDF) [file pone.0249093.s001.pdf]

S1 Table. Baseline characteristics of patients with and without hypotension

**Characteristics**	**Hypotension (n=153)**[a]	**No hypotension (n=198)**[a]	***P* value**
Age, *median (IQR)*, years	70 (60-78)	71 (59-79)	.53
Male, *n (%)*	76/153 (50)	115/198 (59)	.14
Medical history, *n (%)*			
Atrial fibrillation	24/151 (15.9)	53/193 (27.5)	.03
Diabetes mellitus	16/152 (10.5)	33/198 (16.6)	.14
Hypercholesterolemia	76/152 (50.0)	94/197 (47.7)	.52
Hypertension	93/152 (61.2)	120/198 (60.6)	.52
Myocardial infarction	25/149 (16.8)	16/194 (8.2)	.05
Previous stroke	19/153 (12.4)	25/196 (12.8)	.46
Antithrombotic medication, *n (%)*	69/152 (45.4)	85/197 (43.1)	.90
Prestroke mRS score >2, *n (%)*	20/123 (16.3)	15/139 (10.8)	.19
Location occlusion, *n (%)*[b]			
Left hemisphere	83/151 (55.0)	112/197 (56.9)	.68
ICA-C	27/151 (17.9)	31/196 (15.8)	.61
ICA-T	45/151 (29.8)	44/196 (22.4)	.12
M1	94/151 (62.3)	150/196 (76.5)	<.01
M2	60/151 (39.7)	70/196 (35.7)	.72
A1	6/151 (4.0)	7/196 (3.6)	.95
A2	4/151 (2.6)	5/196 (2.6)	.97
Collateral score			.39
Absent collaterals	13/146 (8.9)	17/188 (9.0)	
>0% and ≤50% filling of the occluded area	57/146 (39.0)	81/188 (43.1)	
>50% and <100% filling of the occluded area	59/146 (40.4)	58/188 (30.9)	
100% filling of the occluded area	17/146 (11.6)	32/188 (17.0)	
NIHSS score, *median (IQR)*	17 (12-20)	16 (11-19)	.31
Intravenous thrombolysis, *n (%)*	106/151 (70.2)	139/198 (70.2)	.27
Preintervention MAP, *mean (SD)*, mm Hg	100 (18)	113 (17)	<.001
Time from stroke onset to groin puncture, *median (IQR)*, minutes	217 (170-270)	216 (185-260)	.84

A1, anterior cerebral artery, first segment; A2, anterior cerebral artery, second segment; ICA-C, cervical internal carotid artery; ICA-T, internal carotid artery terminus; M1, middle cerebral artery, first segment; M2, middle cerebral artery, second segment; MAP, mean arterial pressure; mRS, modified Rankin Scale; NIHSS, National Institutes of Health Stroke Scale.

IQR, interquartile range; n, number; SD, standard deviation.

[a]Threshold was set to a mean arterial pressure value of 70 mm Hg.

[b]Sum may not equal 100% due to combined occlusions.
