# Supplementary material for: Hypotension during endovascular treatment under general anesthesia for acute ischemic stroke
Source: PLoS One. 2021 Jun 23;16(6):e0249093. doi: 10.1371/journal.pone.0249093 (PMC8221480; doi:10.1371/journal.pone.0249093)
Supplement: S2 Table — A1, anterior cerebral artery, first segment; A2, anterior cerebral artery, second segment; ICA-C, cervical internal carotid artery; ICA-T, internal carotid artery terminus; M1, middle cerebral artery, first segment; M2, middle cerebral artery, second segment; MAP, mean arterial pressure; mRS, modified Rankin Scale; NIHSS, National Institutes of Health Stroke Scale. IQR, interquartile range; n, number; SD, standard deviation. aThreshold was set to a mean arterial pressure 30% below baseline mean arterial pressure. bSum may not equal 100% due to combined occlusions. (PDF) [file pone.0249093.s002.pdf]

**S2 Table. Baseline characteristics of patients with and without hypotension**

| **Characteristics** | **Hypotension (n=172)**[a] | **No hypotension (n=156)**[a] | ***P* value** |
|---|---|---|---|
| Age, *median (IQR)*, years | 71 (63-79) | 68 (57-78) | .15 |
| Male, *n (%)* | 94/172 (55) | 86/156 (56) | >.99 |
| Medical history, *n (%)* | | | |
| Atrial fibrillation | 33/167 (19.8) | 42/155 (27.1) | .09 |
| Diabetes mellitus | 25/171 (14.6) | 21/156 (13.5) | .61 |
| Hypercholesterolemia | 87/171 (50.9) | 74/155 (47.7) | .51 |
| Hypertension | 105/171 (61.4) | 97/156 (62.2) | .63 |
| Myocardial infarction | 19/165 (11.5) | 18/155 (11.6) | .13 |
| Previous stroke | 21/170 (12.4) | 20/156 (12.8) | .40 |
| Antithrombotic medication, *n (%)* | 76/170 (44.7) | 71/156 (45.5) | .40 |
| Prestroke mRS score >2, *n (%)* | 24/140 (17.1) | 10/102 (9.8) | .11 |
| Location occlusion, *n (%)*[b] | | | |
| Left hemisphere | 101/170 (59.4) | 83/156 (53.2) | .21 |
| ICA-C | 32/170 (18.8) | 24/156 (15.4) | .41 |
| ICA-T | 45/170 (26.5) | 40/156 (25.6) | .87 |
| M1 | 117/170 (68.8) | 115/156 (73.7) | .33 |
| M2 | 69/170 (40.6) | 57/156 (36.5) | .30 |
| A1 | 8/170 (4.7) | 5/156 (3.2) | .32 |
| A2 | 6/170 (3.5) | 3/156 (1.9) | .27 |
| Collateral score | | | .19 |
| Absent collaterals | 14/160 (8.8) | 14/153 (9.2) | |
| >0% and ≤50% filling of the occluded area | 63/160 (39.4) | 68/153 (44.4) | |
| >50% and <100% filling of the occluded area | 60/160 (37.5) | 48/153 (31.4) | |
| 100% filling of the occluded area | 23/160 (14.4) | 23/153 (15.0) | |
| NIHSS score, *median (IQR)* | 17 (12-20) | 16 (11-19) | .21 |
| Intravenous thrombolysis, *n (%)* | 120/170 (70.6) | 107/156 (68.6) | .37 |
| Preintervention MAP, *mean (SD)*, mm Hg | 115 (17) | 99 (17) | <.001 |
| Time from stroke onset to groin puncture, *median (IQR)*, minutes | 215 (180-255) | 216 (182-265) | .63 |

A1, anterior cerebral artery, first segment; A2, anterior cerebral artery, second segment; ICA-C, cervical internal carotid artery; ICA-T, internal carotid artery terminus; M1, middle cerebral artery, first segment; M2, middle cerebral artery, second segment; MAP, mean arterial pressure; mRS, modified Rankin Scale; NIHSS, National Institutes of Health Stroke Scale.

IQR, interquartile range; n, number; SD, standard deviation.

[a]Threshold was set to a mean arterial pressure 30% below baseline mean arterial pressure.

[b]Sum may not equal 100% due to combined occlusions.
