# Supplementary material for: Hypotension during endovascular treatment under general anesthesia for acute ischemic stroke
Source: PLoS One. 2021 Jun 23;16(6):e0249093. doi: 10.1371/journal.pone.0249093 (PMC8221480; doi:10.1371/journal.pone.0249093)
Supplement: S3 Table — CI, confidence interval; OR, odds ratio. aMedian modified Rankin Scale score was 3 (2–6). bOdds ratio per 10 mm Hg*min increase. cThreshold was set to a mean arterial pressure value of 70 mm Hg. dThreshold was set to a mean arterial pressure 30% below baseline mean arterial pressure. eOdds ratio per period increase. fOdds ratio per minute increase. hΔMAP was defined as the difference between baseline MAP and single lowest procedural MAP. (PDF) [file pone.0249093.s003.pdf]

S3 Table. Unadjusted odds ratios for the association between procedural hemodynamics and functional outcome

Variable	OR[a]	95% CI
Area under the threshold[b]		
Absolute threshold[c]	1.000	0.998-1.001
Relative threshold[d]	0.999	0.999-1.000
Occurrence of hypotension		
Absolute threshold[c]	0.64	0.43-0.97
Relative threshold[d]	0.73	0.50-1.07
Number of hypotensive periods[e]		
Absolute threshold[c]	0.89	0.78-1.03
Relative threshold[d]	0.89	0.78-1.01
Total hypotension duration[f]		
Absolute threshold[c]	0.99	0.98-1.00
Relative threshold[d]	0.99	0.98-1.00
ΔMAP[h]	0.987	0.977-0.998

CI, confidence interval; OR, odds ratio.

[a]Median modified Rankin Scale score was 3 (2-6).

[b]Odds ratio per 10 mm Hg*min increase.

[c]Threshold was set to a mean arterial pressure value of 70 mm Hg.

[d]Threshold was set to a mean arterial pressure 30% below baseline mean arterial pressure.

[e]Odds ratio per period increase.

[f]Odds ratio per minute increase.

[h]ΔMAP was defined as the difference between baseline MAP and single lowest procedural MAP.
