# Supplementary material for: Hypotension during endovascular treatment under general anesthesia for acute ischemic stroke
Source: PLoS One. 2021 Jun 23;16(6):e0249093. doi: 10.1371/journal.pone.0249093 (PMC8221480; doi:10.1371/journal.pone.0249093)
Supplement: S4 Table — aThreshold was set to a mean arterial pressure value of 70 mm Hg. bSuccessful reperfusion was defined as modified Thrombolysis In Cerebral Infarction score of ≥2B. cEarly neurologic recovery was defined as National Institutes of Health Stroke Scale score of 0 or 1 within 24 hours postprocedural, or a decrease of 8 points relative to baseline. dSymptomatic intracranial hemorrhage was defined as parenchymal hemorrhage with early neurologic deterioration (an increase of ≥4 points in score on the National Institutes of Health Stroke Scale). (PDF) [file pone.0249093.s004.pdf]

S4 Table. Postprocedural outcome variables of patients with and without hypotension

**Secondary outcomes**	**Hypotension (n=153)**[a]	**No hypotension (n=198)**[a]	***P* value**
Successful reperfusion, *n (%)*[b]	123/153 (80)	172/197 (87)	.14
Early neurologic recovery, *n (%)*[c]	38/144 (26)	43/189 (23)	.64
Symptomatic intracranial hemorrhage, *n (%)*[d]	16/153 (10)	7/198 (3.5)	.02
In-hospital mortality, *n (%)*	19/153 (12)	15/198 (7.6)	.18

[a]Threshold was set to a mean arterial pressure value of 70 mm Hg.
[b]Successful reperfusion was defined as modified Thrombolysis In Cerebral Infarction score of ≥2B.
[c]Early neurologic recovery was defined as National Institutes of Health Stroke Scale score of 0 or 1 within 24 hours postprocedural, or a decrease of 8 points relative to baseline.
[d]Symptomatic intracranial hemorrhage was defined as parenchymal hemorrhage with early neurologic deterioration (an increase of ≥4 points in score on the National Institutes of Health Stroke Scale).
