# Supplementary material for: Hypotension during endovascular treatment under general anesthesia for acute ischemic stroke
Source: PLoS One. 2021 Jun 23;16(6):e0249093. doi: 10.1371/journal.pone.0249093 (PMC8221480; doi:10.1371/journal.pone.0249093)
Supplement: S5 Table — aThreshold was set to a mean arterial pressure 30% below baseline mean arterial pressure. bSuccessful reperfusion was defined as modified Thrombolysis In Cerebral Infarction score of ≥2B. cEarly neurologic recovery was defined as National Institutes of Health Stroke Scale score of 0 or 1 within 24 hours postprocedural, or a decrease of 8 points relative to baseline. dSymptomatic intracranial hemorrhage was defined as parenchymal hemorrhage with early neurologic deterioration (an increase of ≥4 points in score on the National Institutes of Health Stroke Scale). (PDF) [file pone.0249093.s005.pdf]

**S5 Table. Postprocedural outcome variables of patients with and without hypotension**

| **Secondary outcomes** | **Hypotension (n=172)**[a] | **No hypotension (n=156)**[a] | ***P* value** |
|---|---|---|---|
| Successful reperfusion, *n (%)*[b] | 141/172 (82) | 135/155 (87) | .26 |
| Early neurologic recovery, *n (%)*[c] | 45/162 (28) | 30/152 (20) | .09 |
| Symptomatic intracranial hemorrhage, *n (%)*[d] | 12/172 (7.0) | 9/156 (5.8) | .83 |
| In-hospital mortality, *n (%)* | 14/172 (8.1) | 17/156 (11) | .51 |

[a]Threshold was set to a mean arterial pressure 30% below baseline mean arterial pressure.

[b]Successful reperfusion was defined as modified Thrombolysis In Cerebral Infarction score of ≥2B.

[c]Early neurologic recovery was defined as National Institutes of Health Stroke Scale score of 0 or 1 within 24 hours postprocedural, or a decrease of 8 points relative to baseline.

[d]Symptomatic intracranial hemorrhage was defined as parenchymal hemorrhage with early neurologic deterioration (an increase of ≥4 points in score on the National Institutes of Health Stroke Scale).
