# Supplementary material for: Hypotension during endovascular treatment under general anesthesia for acute ischemic stroke
Source: PLoS One. 2021 Jun 23;16(6):e0249093. doi: 10.1371/journal.pone.0249093 (PMC8221480; doi:10.1371/journal.pone.0249093)
Supplement: S6 Table — CI, confidence interval; OR, odds ratio. aSuccessful reperfusion was defined as modified Thrombolysis In Cerebral Infarction score of ≥2B. bEarly neurologic recovery was defined as National Institutes of Health Stroke Scale score of 0 or 1 within 24 hours postprocedural, or a decrease of 8 points relative to baseline. cSymptomatic intracranial hemorrhage was defined as parenchymal hemorrhage with early neurologic deterioration (an increase of ≥4 points in score on the National Institutes of Health Stroke Scale). dThreshold was set to a mean arterial pressure value of 70 mm Hg. eOdds ratio per 10 mm Hg*min increase. fThreshold was set to a mean arterial pressure 30% below baseline mean arterial pressure. (PDF) [file pone.0249093.s006.pdf]

S6 Table. Unadjusted odds ratios for the association between area under the threshold and secondary safety endpoints

	**Successful reperfusion**[a]	**Early neurologic recovery**[b]	**Symptomatic intracranial hemorrhage**[c]	**In-hospital mortality**
Absolute threshold[d]				
**OR**[e]	0.999	1.000	1.003	1.002
**95% CI**	0.997-1.002	0.998-1.002	1.000-1.005	0.999-1.003
Relative threshold[f]				
**OR**[e]	1.000	1.000	1.001	1.000
**95% CI**	0.998-1.000	0.999-1.001	1.001-1.002	1.000-1.001

CI, confidence interval; OR, odds ratio.

[a]Successful reperfusion was defined as modified Thrombolysis In Cerebral Infarction score of ≥2B.

[b]Early neurologic recovery was defined as National Institutes of Health Stroke Scale score of 0 or 1 within 24 hours postprocedural, or a decrease of 8 points relative to baseline.

[c]Symptomatic intracranial hemorrhage was defined as parenchymal hemorrhage with early neurologic deterioration (an increase of ≥4 points in score on the National Institutes of Health Stroke Scale).

[d]Threshold was set to a mean arterial pressure value of 70 mm Hg.

[e]Odds ratio per 10 mm Hg*min increase.

[f]Threshold was set to a mean arterial pressure 30% below baseline mean arterial pressure.
