# Supplementary figures and images for: Hypotension during endovascular treatment under general anesthesia for acute ischemic stroke
Source: PLoS One. 2021 Jun 23;16(6):e0249093. doi: 10.1371/journal.pone.0249093 (PMC8221480; doi:10.1371/journal.pone.0249093)

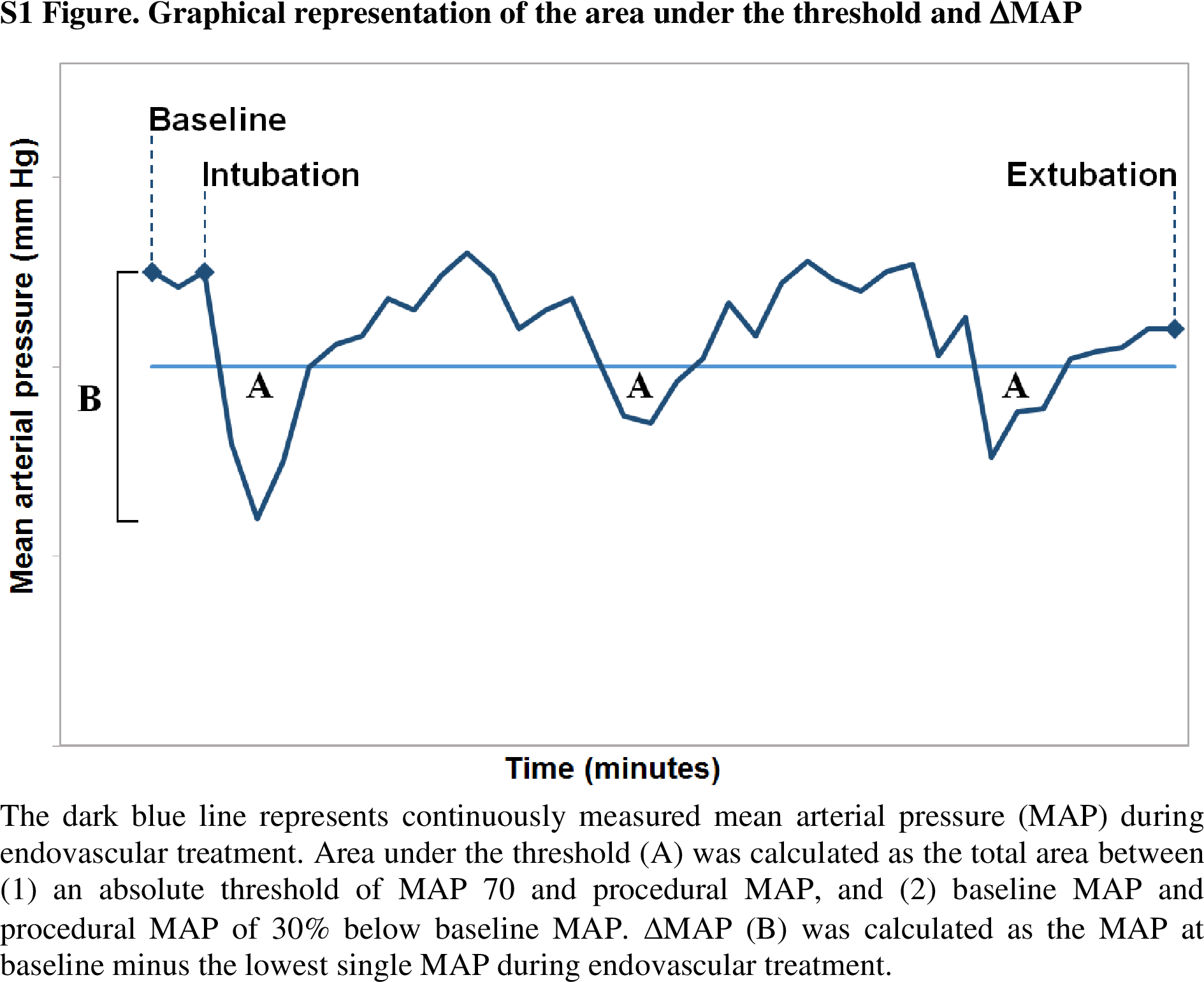

Supplement: S1 Fig — The dark blue line represents continuously measured mean arterial pressure (MAP) during endovascular treatment. Area under the threshold (A) was calculated as the total area between (1) an absolute threshold of MAP 70 and procedural MAP, and (2) baseline MAP and procedural MAP of 30% below baseline MAP. ΔMAP (B) was calculated as the MAP at baseline minus the lowest single MAP during endovascular treatment. (TIF) [file pone.0249093.s007.tif]

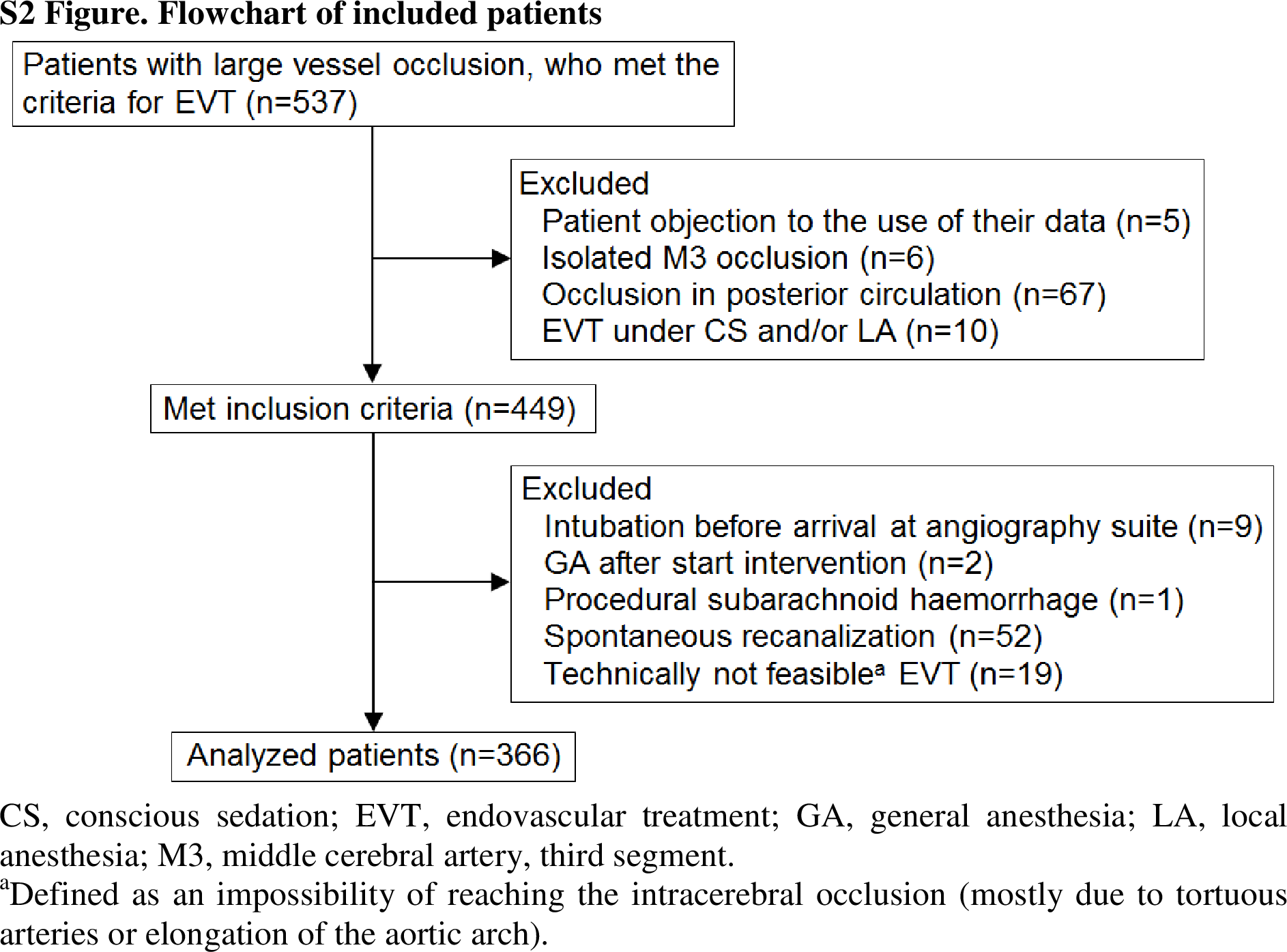

Supplement: S2 Fig — CS, conscious sedation; EVT, endovascular treatment; GA, general anesthesia; LA, local anesthesia; M3, middle cerebral artery, third segment. aDefined as an impossibility of reaching the intracerebral occlusion (mostly due to tortuous arteries or elongation of the aortic arch). (TIF) [file pone.0249093.s008.tif]

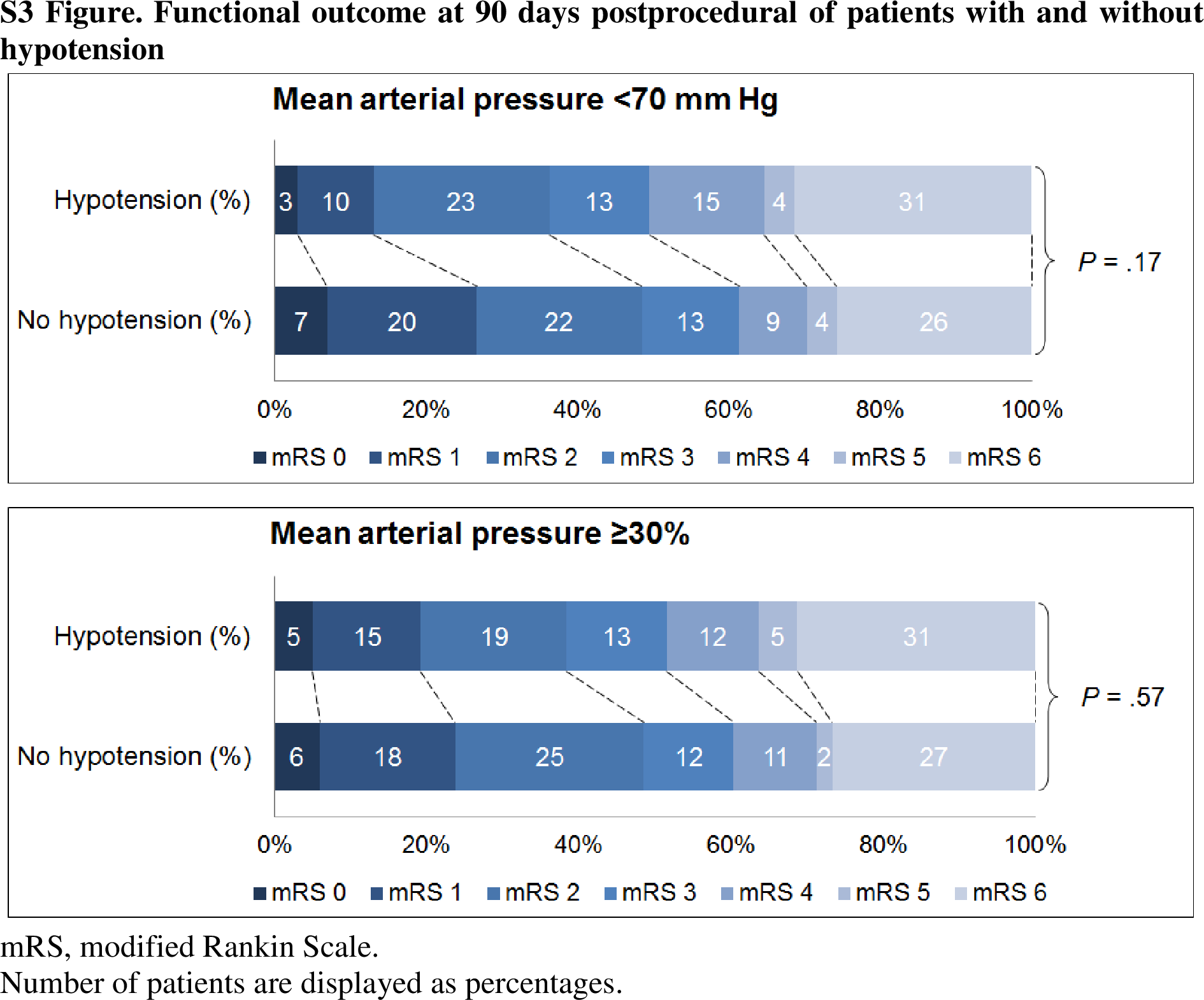

Supplement: S3 Fig — mRS, modified Rankin Scale. Number of patients are displayed as percentages. (TIF) [file pone.0249093.s009.tif]
